# 1,1′-Bicyclo­hexyl-1,1′-diyl 1,1′-biphenyl-2,2′-dicarboxyl­ate

**DOI:** 10.1107/S1600536812018478

**Published:** 2012-05-05

**Authors:** Hoong-Kun Fun, Ching Kheng Quah, Dongdong Wu, Yan Zhang

**Affiliations:** a X-ray Crystallography Unit, School of Physics, Universiti Sains Malaysia, 11800 USM, Penang, Malaysia; b School of Chemistry and Chemical Engineering, Nanjing University, Nanjing 210093, People’s Republic of China

## Abstract

The title compound, C_26_H_28_O_4_, lies about a crystallographic twofold rotation axis. The cyclo­hexane rings adopt a chair conformation. The two benzene rings form a dihedral angle of 40.82 (3)°. No significant intra- or inter­molecular inter­actions are observed in the crystal structure.

## Related literature

For general background to and the biological activity of the title compound, see: Lei *et al.* (2004 ▶); Wu *et al.* (2002 ▶, 2012 ▶); Quideau *et al.* (1996 ▶); Yoshimura *et al.* (2008 ▶). For the stability of the temperature controller used for the data collection, see: Cosier & Glazer (1986 ▶). For standard bond-length data, see: Allen *et al.* (1987 ▶). For ring conformations, see: Cremer & Pople (1975 ▶).
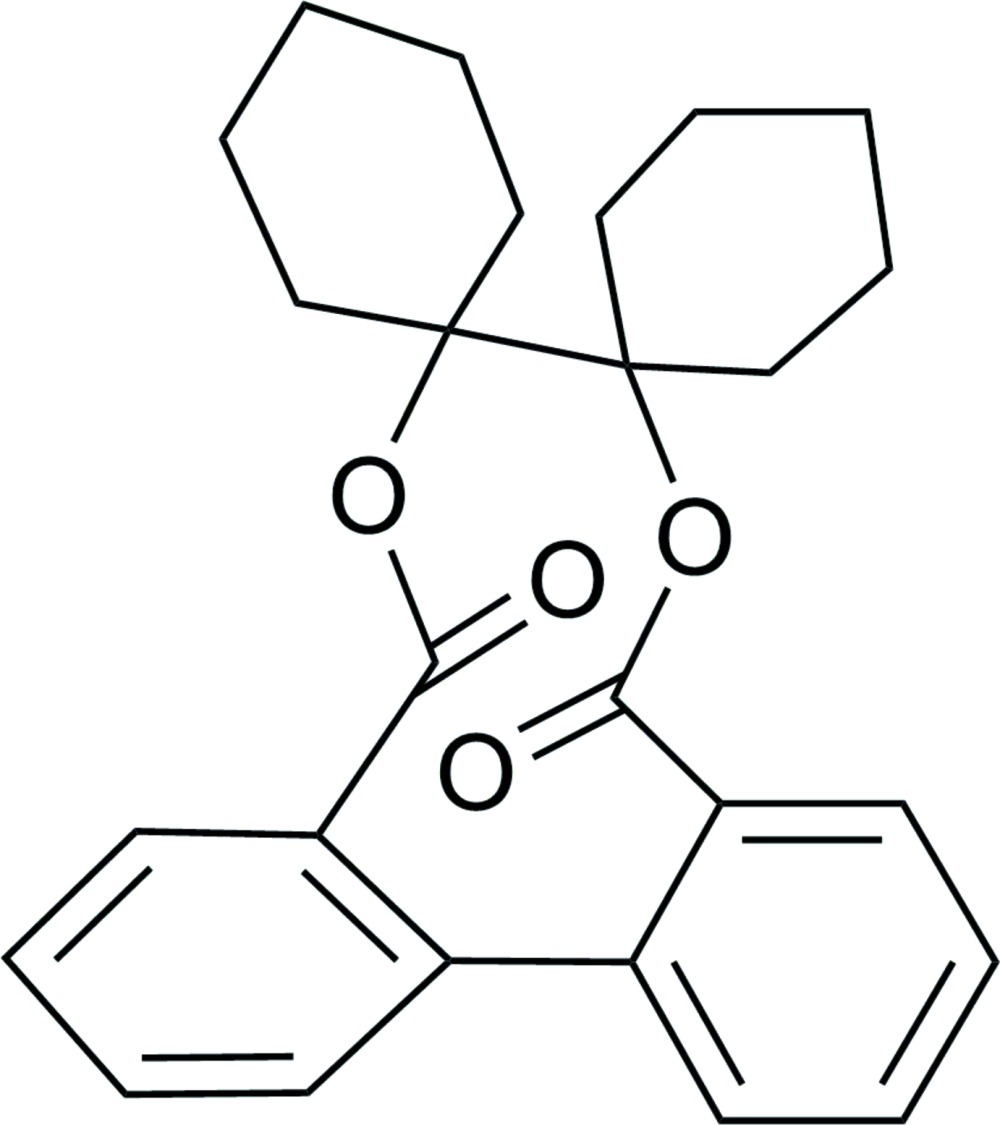


## Experimental

### 

#### Crystal data

C_26_H_28_O_4_
*M* *_r_* = 404.48
Monoclinic, 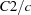

*a* = 16.8289 (7) Å
*b* = 10.5919 (5) Å
*c* = 11.4752 (5) Å
β = 99.967 (1)°
*V* = 2014.58 (15) Å^3^
*Z* = 4
Mo *K*α radiation
μ = 0.09 mm^−1^
*T* = 100 K
0.38 × 0.37 × 0.37 mm

#### Data collection

Bruker SMART APEXII DUO CCD area-detector diffractometer
Absorption correction: multi-scan (*SADABS*; Bruker, 2009 ▶) *T*_min_ = 0.967, *T*_max_ = 0.968
16772 measured reflections
4382 independent reflections
4006 reflections with *I* > 2σ(*I*)
*R*_int_ = 0.019

#### Refinement

*R*[*F* ^2^ > 2σ(*F* ^2^)] = 0.035
*wR*(*F* ^2^) = 0.107
*S* = 1.05
4382 reflections
136 parameters
H-atom parameters constrained
Δρ_max_ = 0.47 e Å^−3^
Δρ_min_ = −0.23 e Å^−3^

### 

Data collection: *APEX2* (Bruker, 2009 ▶); cell refinement: *SAINT* (Bruker, 2009 ▶); data reduction: *SAINT*; program(s) used to solve structure: *SHELXTL* (Sheldrick, 2008 ▶); program(s) used to refine structure: *SHELXTL*; molecular graphics: *SHELXTL*; software used to prepare material for publication: *SHELXTL* and *PLATON* (Spek, 2009 ▶).

## Supplementary Material

Crystal structure: contains datablock(s) global, I. DOI: 10.1107/S1600536812018478/is5122sup1.cif

Structure factors: contains datablock(s) I. DOI: 10.1107/S1600536812018478/is5122Isup2.hkl

Supplementary material file. DOI: 10.1107/S1600536812018478/is5122Isup3.cml

Additional supplementary materials:  crystallographic information; 3D view; checkCIF report

## Figures and Tables

**Fig. 1.**
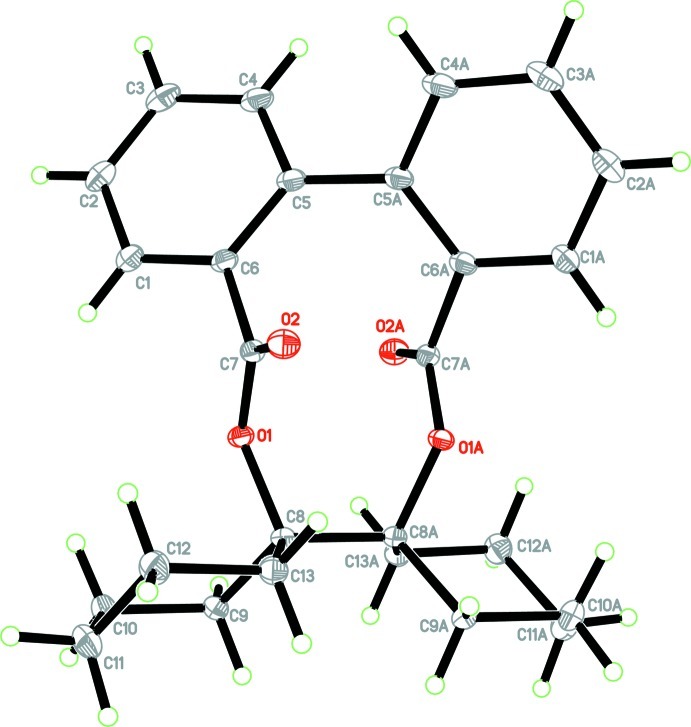
The molecular structure of the title compound showing 50% probability displacement ellipsoids for non-H atoms. Atoms with suffix A were generated by the symmetry code -x + 1, y, -z + 1/2.

## supplementary crystallographic information

### Comment

Biaryl motifs are present in a large number of natural products, dyes, chiral ligands and chiral catalysts (Lei *et al.*, 2004, Wu *et al.*, 2002). Biphenyl-containing medium-sized lactones containing biaryl motif are also important structural core found in many biologically active natural products, such as ellagitannins family (Quideau *et al.*, 1996). Flavonol glucuronides and C-glucosidic ellagitannins which were isolated from the leaves of *Melaleuca squarrosa* shown *in vitro* antioxidant activity that can be evaluated by DPPH radical in the usual way (Yoshimura *et al.*, 2008). The crystal structures of 5,10-dioxo-5,7,8,10-tetrahydrodibenzo[*f*,*h*] [1,4]dioxecin-7-yl benzoate, 7-methyl-8-phenyl-7,8-dihydrodibenzo [*f*,*h*][1,4]dioxecine-5,10-dione and 7-phenyl-7,8-dihydro-[1,4]dioxecino[7,6-b:8,9-b'] dipyridine-5,10-dione (Wu *et al.*, 2012) have been reported. Due to the importance of the biphenyl-containing medium-sized rings, we report here the crystal structure of the title compound in this paper.

The title compound, Fig. 1, lies about a crystallographic twofold axis generated by the symmetry code -x+1, y, -z+1/2. The cyclohexane ring (C8–C13) adopts a chair conformation with puckering parameters (Cremer & Pople, 1975) Q = 0.5752 (7) Å, Θ = 177.40 (7)° and φ = 114.1 (14)°. The two benzene rings (C1–C6 & C1A–C6A) form a dihedral angle of 40.82 (3)°. Bond lengths (Allen *et al.*, 1987) and angles are within normal ranges. There are no significant hydrogen bonds observed in this compound.

### Experimental

The title compound was the product from the photooxidation between 2,3- dispirocyclohexyl-2,3-dihydrophenanthro[9,10-b][1,4]dioxine and oxygen. The compound was purified by flash column chromatography with ethyl acetate/petroleum ether (1:10) as eluents. X-ray quality crystals of the title compound, were obtained from slow evaporation of an acetone and petroleum ether solution (1:10).

### Refinement

All H atoms were positioned geometrically and refined using a riding model with C—H = 0.93 or 0.97 Å, and with *U*_iso_(H) = 1.2*U*_eq_(C).

### Crystal data

**Table:**

C_26_H_28_O_4_	*F*(000) = 864
*M**_r_* = 404.48	*D*_x_ = 1.334 Mg m^−^^3^
Monoclinic, *C*2/*c*	Mo *K*α radiation, λ = 0.71073 Å
Hall symbol: -C 2yc	Cell parameters from 9620 reflections
*a* = 16.8289 (7) Å	θ = 4.2–35.0°
*b* = 10.5919 (5) Å	µ = 0.09 mm^−^^1^
*c* = 11.4752 (5) Å	*T* = 100 K
β = 99.967 (1)°	Block, colourless
*V* = 2014.58 (15) Å^3^	0.38 × 0.37 × 0.37 mm
*Z* = 4	

### Data collection

**Table:**

Bruker SMART APEXII DUO CCD area-detector diffractometer	4382 independent reflections
Radiation source: fine-focus sealed tube	4006 reflections with *I* > 2σ(*I*)
Graphite monochromator	*R*_int_ = 0.019
φ and ω scans	θ_max_ = 35.0°, θ_min_ = 4.2°
Absorption correction: multi-scan (*SADABS*; Bruker, 2009)	*h* = −21→27
*T*_min_ = 0.967, *T*_max_ = 0.968	*k* = −17→12
16772 measured reflections	*l* = −18→18

### Refinement

**Table:**

Refinement on *F*^2^	Primary atom site location: structure-invariant direct methods
Least-squares matrix: full	Secondary atom site location: difference Fourier map
*R*[*F*^2^ > 2σ(*F*^2^)] = 0.035	Hydrogen site location: inferred from neighbouring sites
*wR*(*F*^2^) = 0.107	H-atom parameters constrained
*S* = 1.05	*w* = 1/[σ^2^(*F*_o_^2^) + (0.0621*P*)^2^ + 0.7551*P*] where *P* = (*F*_o_^2^ + 2*F*_c_^2^)/3
4382 reflections	(Δ/σ)_max_ = 0.001
136 parameters	Δρ_max_ = 0.47 e Å^−^^3^
0 restraints	Δρ_min_ = −0.23 e Å^−^^3^

### Special details

**Table:**

Experimental. The crystal was placed in the cold stream of an Oxford Cryosystems Cobra open-flow nitrogen cryostat (Cosier & Glazer, 1986) operating at 100.0 (1) K.
Geometry. All esds (except the esd in the dihedral angle between two l.s. planes) are estimated using the full covariance matrix. The cell esds are taken into account individually in the estimation of esds in distances, angles and torsion angles; correlations between esds in cell parameters are only used when they are defined by crystal symmetry. An approximate (isotropic) treatment of cell esds is used for estimating esds involving l.s. planes.
Refinement. Refinement of F^2^ against ALL reflections. The weighted R-factor wR and goodness of fit S are based on F^2^, conventional R-factors R are based on F, with F set to zero for negative F^2^. The threshold expression of F^2^ > 2sigma(F^2^) is used only for calculating R-factors(gt) etc. and is not relevant to the choice of reflections for refinement. R-factors based on F^2^ are statistically about twice as large as those based on F, and R- factors based on ALL data will be even larger.

### Fractional atomic coordinates and isotropic or equivalent isotropic displacement parameters (Å^2^)

**Table:**

	*x*	*y*	*z*	*U*_iso_*/*U*_eq_	
O1	0.42941 (3)	0.08237 (4)	0.17817 (4)	0.01098 (9)	
O2	0.41218 (3)	0.19432 (4)	0.34309 (4)	0.01462 (9)	
C1	0.35947 (4)	0.30612 (6)	0.05831 (5)	0.01532 (11)	
H1A	0.3278	0.2356	0.0346	0.018*	
C2	0.34982 (4)	0.41537 (6)	−0.01047 (6)	0.01852 (12)	
H2A	0.3112	0.4184	−0.0790	0.022*	
C3	0.39828 (4)	0.51981 (6)	0.02401 (6)	0.01974 (12)	
H3A	0.3915	0.5936	−0.0205	0.024*	
C4	0.45706 (4)	0.51343 (6)	0.12541 (6)	0.01746 (11)	
H4A	0.4903	0.5828	0.1463	0.021*	
C5	0.46751 (3)	0.40532 (5)	0.19691 (5)	0.01327 (10)	
C6	0.41629 (3)	0.30170 (5)	0.16241 (5)	0.01239 (10)	
C7	0.41890 (3)	0.18771 (5)	0.24002 (5)	0.01133 (10)	
C8	0.45225 (3)	−0.04149 (5)	0.23283 (5)	0.01039 (9)	
C9	0.42275 (3)	−0.13489 (5)	0.13258 (5)	0.01332 (10)	
H9A	0.4472	−0.1135	0.0646	0.016*	
H9B	0.4405	−0.2192	0.1580	0.016*	
C10	0.33088 (4)	−0.13507 (6)	0.09545 (6)	0.01723 (11)	
H10A	0.3136	−0.0540	0.0606	0.021*	
H10B	0.3156	−0.1995	0.0357	0.021*	
C11	0.28797 (4)	−0.16018 (7)	0.19990 (6)	0.01998 (12)	
H11A	0.2992	−0.2457	0.2283	0.024*	
H11B	0.2302	−0.1520	0.1746	0.024*	
C12	0.31654 (4)	−0.06665 (6)	0.29988 (6)	0.01688 (11)	
H12A	0.2913	−0.0868	0.3675	0.020*	
H12B	0.3003	0.0181	0.2739	0.020*	
C13	0.40844 (3)	−0.07157 (5)	0.33654 (5)	0.01320 (10)	
H13A	0.4241	−0.1551	0.3668	0.016*	
H13B	0.4250	−0.0113	0.3998	0.016*	

### Atomic displacement parameters (Å^2^)

**Table:**

	*U*^11^	*U*^22^	*U*^33^	*U*^12^	*U*^13^	*U*^23^
O1	0.01283 (17)	0.00808 (16)	0.01182 (17)	0.00106 (12)	0.00151 (13)	0.00043 (12)
O2	0.01673 (19)	0.01344 (19)	0.01440 (18)	0.00038 (14)	0.00467 (14)	−0.00096 (13)
C1	0.0149 (2)	0.0138 (2)	0.0166 (2)	0.00300 (17)	0.00097 (18)	0.00128 (17)
C2	0.0185 (3)	0.0183 (3)	0.0184 (3)	0.0063 (2)	0.0021 (2)	0.0042 (2)
C3	0.0218 (3)	0.0154 (3)	0.0227 (3)	0.0061 (2)	0.0056 (2)	0.0066 (2)
C4	0.0194 (3)	0.0105 (2)	0.0231 (3)	0.00200 (18)	0.0056 (2)	0.00334 (19)
C5	0.0143 (2)	0.0090 (2)	0.0169 (2)	0.00124 (16)	0.00385 (17)	0.00066 (16)
C6	0.0132 (2)	0.0093 (2)	0.0148 (2)	0.00178 (16)	0.00282 (17)	0.00097 (16)
C7	0.0099 (2)	0.0095 (2)	0.0145 (2)	0.00032 (15)	0.00178 (16)	−0.00049 (15)
C8	0.0114 (2)	0.00798 (19)	0.0115 (2)	−0.00010 (15)	0.00114 (15)	0.00068 (15)
C9	0.0140 (2)	0.0106 (2)	0.0143 (2)	−0.00026 (16)	−0.00064 (17)	−0.00214 (16)
C10	0.0144 (2)	0.0169 (2)	0.0187 (3)	−0.00241 (18)	−0.00193 (19)	−0.00231 (19)
C11	0.0146 (2)	0.0195 (3)	0.0248 (3)	−0.0057 (2)	0.0004 (2)	0.0012 (2)
C12	0.0132 (2)	0.0189 (3)	0.0191 (2)	−0.00175 (18)	0.00416 (19)	0.00251 (19)
C13	0.0133 (2)	0.0133 (2)	0.0132 (2)	−0.00114 (16)	0.00258 (17)	0.00192 (16)

### Geometric parameters (Å, º)

**Table:**

O1—C7	1.3503 (7)	C8—C13	1.5379 (8)
O1—C8	1.4760 (7)	C8—C8^i^	1.5872 (11)
O2—C7	1.2095 (7)	C9—C10	1.5310 (8)
C1—C2	1.3942 (8)	C9—H9A	0.9700
C1—C6	1.3958 (8)	C9—H9B	0.9700
C1—H1A	0.9300	C10—C11	1.5258 (10)
C2—C3	1.3906 (10)	C10—H10A	0.9700
C2—H2A	0.9300	C10—H10B	0.9700
C3—C4	1.3923 (10)	C11—C12	1.5290 (10)
C3—H3A	0.9300	C11—H11A	0.9700
C4—C5	1.4020 (8)	C11—H11B	0.9700
C4—H4A	0.9300	C12—C13	1.5318 (8)
C5—C6	1.4094 (8)	C12—H12A	0.9700
C5—C5^i^	1.4896 (12)	C12—H12B	0.9700
C6—C7	1.4964 (8)	C13—H13A	0.9700
C8—C9	1.5332 (8)	C13—H13B	0.9700
			
C7—O1—C8	124.00 (4)	C10—C9—H9A	109.0
C2—C1—C6	120.51 (6)	C8—C9—H9A	109.0
C2—C1—H1A	119.7	C10—C9—H9B	109.0
C6—C1—H1A	119.7	C8—C9—H9B	109.0
C3—C2—C1	119.61 (6)	H9A—C9—H9B	107.8
C3—C2—H2A	120.2	C11—C10—C9	111.97 (5)
C1—C2—H2A	120.2	C11—C10—H10A	109.2
C2—C3—C4	119.74 (6)	C9—C10—H10A	109.2
C2—C3—H3A	120.1	C11—C10—H10B	109.2
C4—C3—H3A	120.1	C9—C10—H10B	109.2
C3—C4—C5	121.85 (6)	H10A—C10—H10B	107.9
C3—C4—H4A	119.1	C10—C11—C12	110.27 (5)
C5—C4—H4A	119.1	C10—C11—H11A	109.6
C4—C5—C6	117.58 (6)	C12—C11—H11A	109.6
C4—C5—C5^i^	118.60 (4)	C10—C11—H11B	109.6
C6—C5—C5^i^	123.81 (4)	C12—C11—H11B	109.6
C1—C6—C5	120.62 (5)	H11A—C11—H11B	108.1
C1—C6—C7	118.79 (5)	C11—C12—C13	110.82 (5)
C5—C6—C7	120.51 (5)	C11—C12—H12A	109.5
O2—C7—O1	127.21 (5)	C13—C12—H12A	109.5
O2—C7—C6	122.49 (5)	C11—C12—H12B	109.5
O1—C7—C6	110.30 (5)	C13—C12—H12B	109.5
O1—C8—C9	103.18 (4)	H12A—C12—H12B	108.1
O1—C8—C13	112.87 (4)	C12—C13—C8	112.14 (5)
C9—C8—C13	108.10 (4)	C12—C13—H13A	109.2
O1—C8—C8^i^	106.46 (3)	C8—C13—H13A	109.2
C9—C8—C8^i^	111.63 (4)	C12—C13—H13B	109.2
C13—C8—C8^i^	114.10 (5)	C8—C13—H13B	109.2
C10—C9—C8	112.94 (5)	H13A—C13—H13B	107.9
			
C6—C1—C2—C3	1.18 (10)	C1—C6—C7—O1	56.26 (7)
C1—C2—C3—C4	1.34 (10)	C5—C6—C7—O1	−126.93 (5)
C2—C3—C4—C5	−2.06 (10)	C7—O1—C8—C9	157.39 (5)
C3—C4—C5—C6	0.24 (9)	C7—O1—C8—C13	40.97 (7)
C3—C4—C5—C5^i^	179.20 (6)	C7—O1—C8—C8^i^	−84.97 (6)
C2—C1—C6—C5	−3.04 (9)	O1—C8—C9—C10	−64.78 (6)
C2—C1—C6—C7	173.76 (5)	C13—C8—C9—C10	54.98 (6)
C4—C5—C6—C1	2.29 (8)	C8^i^—C8—C9—C10	−178.73 (4)
C5^i^—C5—C6—C1	−176.60 (6)	C8—C9—C10—C11	−55.23 (7)
C4—C5—C6—C7	−174.45 (5)	C9—C10—C11—C12	54.07 (7)
C5^i^—C5—C6—C7	6.65 (10)	C10—C11—C12—C13	−55.70 (7)
C8—O1—C7—O2	−13.97 (9)	C11—C12—C13—C8	58.70 (6)
C8—O1—C7—C6	166.08 (5)	O1—C8—C13—C12	56.62 (6)
C1—C6—C7—O2	−123.70 (6)	C9—C8—C13—C12	−56.84 (6)
C5—C6—C7—O2	53.11 (8)	C8^i^—C8—C13—C12	178.33 (4)

Symmetry code: (i) −*x*+1, *y*, −*z*+1/2.
